# Molecular connectivity indices for modeling the critical micelle concentration of cationic (chloride) Gemini surfactants

**DOI:** 10.1007/s00396-016-3979-3

**Published:** 2016-11-17

**Authors:** Anna Mozrzymas

**Affiliations:** Department of Physics and Biophysics, Wrocław University of Environmental and Life Sciences, ul. Norwida 25, 50-375 Wrocław, Poland

**Keywords:** Chloride gemini surfactants, QSPR, Cmc, Molecular connectivity indices

## Abstract

The molecular connectivity indices were used to derive the simple model relating the critical micelle concentration of cationic (chloride) gemini surfactants to their structure. One index was selected as the best to describe the effect of the structure of investigated compounds on critical micelle concentration consistent with the experimental results. This index encodes the information about molecular size, the branches, and also the information about heteroatoms. The selected model can be helpful in designing novel chloride gemini surfactants.

## Introduction

The quantitative structure-property relationship (QSPR) studies use the statistical models to estimate the various properties of the chemical compounds from its molecular structure [1–14]. In QSPR studies, the structure is often represented by different structural descriptors. Among the various structural parameters applied to QSPR analysis, the topological indices are often used in modeling physical, chemical, or biological properties [5–14]. The first applications of topological indices in structure-property relationship studies was proposed by Wiener in 1947 [15] and later in the 1975 by Randic[16]. The generalization of the Randic index are the Kier and Hall molecular connectivity indices [10]. The molecular connectivity indices contain considerable information about the structure of the molecule. Kier and Hall [10]widely described the information encoded by molecular connectivity indices especially on thetopological but also the electronic properties of the molecule.

Gemini surfactants consist of two hydrophobic tails and two hydrophilic heads connected by the spacer group. Due to the binding together of two conventional surfactant molecules by the spacer, these compounds have very good properties in aqueous solution. The cmc values of these surfactants are significantly lower than those of the corresponding monomeric counterparts.

In the previous paper [12], the QSPR study was performed to derive the model which relates the critical micelle concentration of gemini surfactants to their structure. The relationship was developed for a set of 21 cationic (bromide) gemini surfactants employing the molecular connectivity indices only. The previous model contains the second-order molecular connectivity index which, as was suggested in [12], probably encodes the information about the flexibility.

In the present study, the 4models were derived. The relationships were developed for a set of 23 cationic (chloride) gemini surfactants also employing only the molecular connectivity indices. Just as in the previous study [12], the present models were derived for the molecules of various structures, i.e., the effect of all groups of the molecule on cmc value was taken into account. The structure of the investigated compounds are quite different from the previous bromides. Also, the test compounds differ in structure from previously studied compounds. The present study confirms that the one-descriptor model which best estimates the cmc values is that which contains the second-order molecular connectivity index, but the further analysis showed that the model which contains the first-order valence molecular connectivity index better describes the changes of cmc values of cationic (chloride) gemini surfactants caused by structure modification.

## Materials and methods

### Dataset

**Fig. 1:**
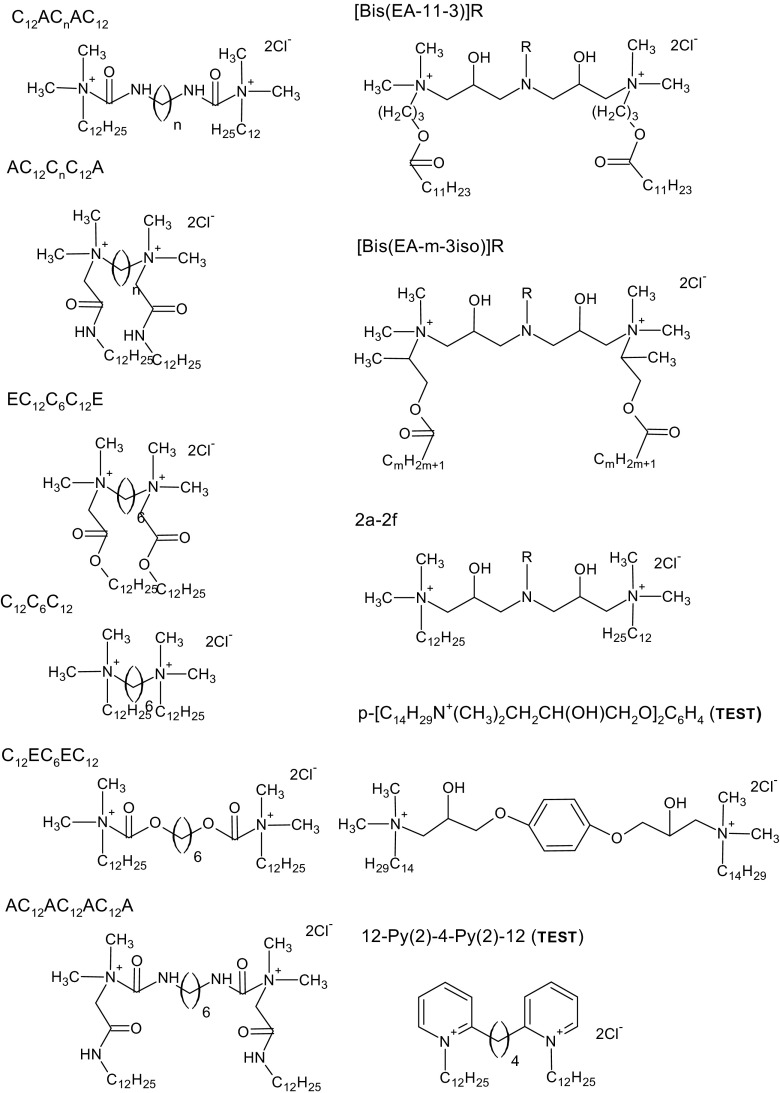
Structures of investigated compounds and their abbreviations

The data set contains only gemini surfactants with chlorides as counterions. The compounds were chosen to contain gemini surfactants with medium and long spacer length. The chemical structures of the investigated compounds along with their abbreviations are presented in Fig. 1. The data set contains 23 compounds of training set and 2test compounds. The chemical structures of the surfactants and the experimental values of cmc were taken from literature [17–21].

### Molecular connectivity indices

Just as in the previous papers [11–13], the structure of the molecule is represented by Kier and Hall’s molecular connectivity indices.

The *m*th order molecular connectivity index is defined [10] by

1 $$ {}^m\chi_k={\displaystyle \sum_{j=1}^{n_m}{\displaystyle \prod_{i=1}^{m+1}{\left({\delta}_i\right)}_j^{-0.5}}} $$

where *δ*_*i*_ is a connectivity degree, i.e., the number of non-hydrogen atoms to which the *i*th non-hydrogen atom is bonded;*m* is the order of the connectivity index;*k* denotes the type of the fragment of the molecule, for example: path (p), cluster (c), and path-cluster (pc); and*n*_*m*_ is the number of fragments of type *k* and order *m*.

For molecules with the heteroatoms, the valence connectivity degree has been defined [10] as

2 $$ {\delta}_i^{\nu }=\frac{Z_i^{\nu }-{h}_i}{Z_i-{Z}_i^{\nu }-1} $$

where ${Z}_i^{\nu }$ is the number of valence electrons in the *i*th atom, *h*_*i*_ is the number of hydrogen atoms connected to the *i*th atom, and *Z*_*i*_ is the number of all electrons in the *i*th atom.

The replacement *δ*_*i*_ by ${\delta}_i^{\nu }$ defines the valence molecular connectivity index ${}^m\chi_k^{\nu }$.

An example of calculations of molecular connectivity indices for one of the investigated gemini surfactants is presented in Appendix 1.

The molecular connectivity indices contain considerable information about the molecule. The low-order molecular connectivity indices include information about atoms and molecular size while cluster and path/cluster molecular connectivity indices include structure information about branch point and branch point environment; the valence indices add information about heteroatoms [10, 22]. For example, ^0^ *χ* ^*ν*^ index includes information about heteroatoms contained in the molecule, ^1^ *χ* and ^1^ *χ* ^*ν*^ indices contain the information about molecular volume and molecular surface area; additionally, the ^1^ *χ* ^*ν*^adds information about heteroatoms but ${}^3\chi_c^{\nu }$ index contains the information about the number of branches and their heteroatoms [10].

### Statistics

The least squares method was used to generate the formula expressing the relationship between the logcmc and the molecular connectivity indices. In order to test the quality of the derived equation, three statistical parameters were used: a coefficient of determination (*r* ^2^), a correlation coefficient (*r*), a Fisher ratio (*F*), and a standard deviation (*s*). The best relationship is that which has possibly the highest values of *r* ^2^,*r*, and *F*and simultaneously the lowest value of *s*.

In the case of the simple linear least-squares model, the values of statistical parameters may be calculated using the following formulas [10]:

3 $$ \hbox{-} \mathrm{the}\ \mathrm{coefficient}\ \mathrm{of}\ \mathrm{determination}:r{}^2=\frac{{\displaystyle \sum {\left({y}_i\left(\mathrm{cal}\right)-\overline{y}\right)}^2}}{{\displaystyle \sum {\left({y}_i\left( \exp \right)-\overline{y}\right)}^2}} $$

where *y*_*i*_(exp)—the experimental value of the property, *y*_*i*_(cal)—the calculated value of the property and $\overline{y}=\frac{1}{n}{\displaystyle \sum_{i=1}^n{y}_i}$,

- the correlation coefficient (*r*) can be obtained from Eq. (3) as a square root of the coefficient of determination. Notice that this definition of *r*, in agreement with ref. [10], does not correspond to the standard definition of Pearson’s linear correlation coefficient, although it has a similar meaning.

4 $$ \hbox{-} \mathrm{the}\ \mathrm{Fisher}\ \mathrm{ratio}:F=\left(n-2\right)\cdot \frac{r^2}{\left(1-{r}^2\right)} $$

5 $$ \hbox{-} \mathrm{the}\ \mathrm{standard}\ \mathrm{deviation}\ \mathrm{of}\ \mathrm{the}\ \mathrm{fit}:s=\sqrt{\frac{{\displaystyle \sum {\left({y}_i\left( \exp \right)-{y}_i\left(\mathrm{cal}\right)\right)}^2}}{n-2}} $$

where *n* is the number of compounds in the data set,

6 $$ \hbox{-} \mathrm{the}\ \mathrm{residual}\ for\ \mathrm{compound}\ i:{\varDelta}_i={y}_i\left( \exp \right)-{y}_i\left(\mathrm{cal}\right) $$

## Results and discussion

The values of the molecular connectivity indices along with the experimental logcmc values for the training set are listed in Table 1.

**Table 1:** Experimental logcmcvalues [17–19] and values of molecular connectivity indices

Compound	logcmc^a^	^0^ *χ*	^1^ *χ*	^2^ *χ*	^3^ *χ*_*c*_	^4^ *χ*_*pc*_	^0^ *χ* ^*ν*^	^1^ *χ* ^*ν*^	^2^ *χ* ^*ν*^	${}^3\chi_c^{\nu }$	${}^4\chi_{pc}^{\nu }$
bis(EA-11-3)C5	−4.31158	40.342595	25.83567	21.71634	3.77304	4.01125	37.06539	22.87411	17.76306	2.693502	2.6531996
bis(EA-11-3)C6	−4.34969	41.049702	26.33567	22.069896	3.77304	4.01125	37.772499	23.37411	18.11661	2.693502	2.6531996
bis(EA-11-3)C8	−4.39041	42.46392	27.33567	22.777	3.77304	4.01125	39.18671	24.37411	18.82372	2.693502	2.6531996
bis(EA-9-3iso)C6	−4.07469	38.54755	24.17711	20.76965	3.86803	6.47149	35.270345	21.22925	16.85702	2.77846	4.74458
bis(EA-9-3iso)C8	−4.10568	39.96176	25.17711	21.47676	3.86803	6.47149	36.68456	22.22925	17.56413	2.77846	4.74458
bis(EA-11-3iso)C5	−4.20204	40.66887	25.67711	21.83031	3.86803	6.47149	37.39167	22.72925	17.91768	2.77846	4.74458
bis(EA-11-3iso)C6	−4.22841	41.37598	26.17711	22.18386	3.86803	6.47149	38.09877	23.22925	18.27124	2.77846	4.74458
bis(EA-11-3iso)C8	−4.266803	42.79019	27.17711	22.89097	3.86803	6.47149	39.51299	24.22925	18.97834	2.77846	4.74458
C_12_AC_2_AC_12_	−3.02687	28.53948	18.16987	14.92768	2.509202	4.85064	26.68149	16.39809	12.64503	1.80341	2.18671
C_12_AC_6_AC_12_	−3.07058	31.367904	20.16987	16.34189	2.509202	4.85064	29.50991	18.39809	14.05924	1.80341	2.18671
C_12_AC_12_AC_12_	−3.65758	35.61054	23.16987	18.46321	2.509202	4.85064	33.75255	21.39809	16.18056	1.80341	2.18671
C_12_C_6_C_12_^b^	−2.88606	26.79899	17.32843	14.1066	2.41421	2.41421	26.69342	16.96798	13.544065	2.15934	2.15934
C_12_EC_6_EC_12_	−3.11351	31.367904	20.16987	16.34189	2.509202	4.85064	29.32641	18.17658	13.82412	1.78666	2.03045
AC_12_C_6_C_12_A	−3.46852	32.78212	21.11612	17.50965	2.99156	2.88963	30.92413	19.29044	15.11298	2.30368	2.00972
AC_12_C_12_C_12_A	−3.79588	37.02476	24.11612	19.63097	2.99156	2.88963	35.16677	22.29044	17.23431	2.30368	2.00972
EC_12_C_6_C_12_E	−3.49485	32.78212	21.11612	17.50965	2.99156	2.88963	30.74062	19.06893	14.85402	2.27719	1.97915
AC_12_AC_6_AC_12_A	−3.60206	37.35103	23.95756	19.74494	3.08655	5.34987	33.74062	20.72055	15.62816	1.94775	2.09135
2a (R = C_2_H_5_)	−3.12843	30.82393	19.54797	16.55666	3.19569	3.25454	29.48265	18.27316	14.70763	2.57565	2.53284
2b (R = C_3_H_7_)	−3.204815	31.53104	20.04797	16.93708	3.19569	3.19475	30.18976	18.77316	15.11501	2.57565	2.48653
2c (R = C_4_H_9_)	−3.25104	32.23815	20.54797	17.29064	3.19569	3.19475	30.896865	19.27316	15.46856	2.57565	2.48653
2d (R = C_5_H_11_)	−3.40561	32.94525	21.04797	17.64419	3.19569	3.19475	31.60397	19.77316	15.82212	2.57565	2.48653
2e (R = C_6_H_13_)	−3.50169	33.65236	21.54797	17.997745	3.19569	3.19475	32.31108	20.27316	16.17567	2.57565	2.48653
2f (R = C_8_H_17_)	−3.598599	35.06657	22.54797	18.70485	3.19569	3.19475	33.72529	21.27316	16.882775	2.57565	2.48653

^a^Thecmc values were measured in pure water at 25 °C

^b^For this compound the values of molecular connectivity indices were taken from [12]

Basing on the data contained in Table 1, the correlation formulas containing one index were derived (Step 1). All values of statistical parameters for the relationships obtained in the first step are listed in Table 2.

**Table 2:** Values ofstatistical parameters for Step 1

Index	^0^ *χ*	^1^ *χ*	^2^ *χ*	^3^ *χ*_*c*_	^4^ *χ*_*pc*_	^0^ *χ* ^*ν*^	^1^ *χ* ^*ν*^	^2^ *χ* ^*ν*^	${}^3\chi_c^{\nu }$	${}^4\chi_{pc}^{\nu }$
*r*	0.978	0.976	0.982	0.864	0.517	0.975	0.957	0.946	0.656	0.636
*F*	465.827	414.171	563.629	61.856	7.658	403.639	231.364	177.729	15.832	14.268
*s*	0.104	0.110	0.095	0.252	0.429	0.111	0.144	0.163	0.378	0.387

As follows from Table 2, the highest values of *r* and *F*andthe lowest value of *s* are for the relationship containing the second-order molecular connectivity index (^2^ *χ*). The inspection of data contained in Table 2 suggests that ^0^ *χ*, ^1^ *χ*, and ^0^ *χ* ^*ν*^ indices also correlate well with the logcmc values. Table 3 shows the correlations between all the indices appearing in Table 1.

**Table 3:** Correlation matrix

	^0^ *χ*	^1^ *χ*	^2^ *χ*	^3^ *χ*_*c*_	^4^ *χ*_*pc*_	^0^ *χ* ^*ν*^	^1^ *χ* ^*ν*^	^2^ *χ* ^*ν*^	${}^3\chi_c^{\nu }$	${}^4\chi_{pc}^{\nu }$
^0^ *χ*	1.000	0.997	0.997	0.848	0.590	0.992	0.974	0.952	0.607	0.650
^1^ *χ*	**0.997**	1.000	0.992	0.816	0.551	0.993	0.982	0.952	0.574	0.598
^2^ *χ*	**0.997**	**0.992**	1.000	0.881	0.568	0.991	0.970	0.959	0.658	0.664
^3^ *χ*_*c*_	0.848	0.816	0.881	1.000	0.473	0.846	0.799	0.860	0.903	0.767
^4^ *χ*_*pc*_	0.590	0.551	0.568	0.473	1.000	0.525	0.443	0.406	0.161	0.799
^0^ *χ* ^*ν*^	**0.992**	**0.993**	**0.991**	0.846	0.525	1.000	0.992	0.981	0.647	0.637
^1^ *χ* ^*ν*^	**0.974**	**0.982**	**0.970**	0.799	0.443	**0.992**	1.000	0.985	0.621	0.562
^2^ *χ* ^*ν*^	0.952	0.952	0.959	0.860	0.406	**0.981**	**0.985**	1.000	0.740	0.621
${}^3\chi_c^{\nu }$	0.607	0.574	0.658	0.903	0.161	0.647	0.621	0.740	1.000	0.660
${}^4\chi_{pc}^{\nu }$	0.650	0.598	0.664	0.767	0.799	0.637	0.562	0.621	0.660	1.000

The bold values mean high correlation

Two indices with *r* ≥ 0.97 are highly correlated, those with 0.90 ≤ *r* < 0.97 are appreciably correlated, the indices with 0.50 ≤ *r* < 0.89 are weakly correlated, and those with *r* < 0.50 are not correlated. As follows from the correlation matrix, there are 12pairs of highly correlated indices, among them, the pairs of ^0^ *χ*and^2^ *χ* indices with value of correlation coefficient 0.997. Because of, the ^0^ *χ* and^2^ *χ* indices carry similar structural information related to the changes of molecular structure, i.e., the values of these indices increase with the increase of atoms and branches in the molecule [10];therefore, we can ignore the ^0^ *χ* index in further considerations. The remaining indices which highly correlate with logcmc values, namely,^2^ *χ*, ^1^ *χ*, and ^0^ *χ* ^*ν*^ indices also highly correlate to each other (Table 3), but they contain somewhat different structure information; especially, their values vary with changes in molecular structure. The first-order molecular connectivity index (^1^ *χ*) decreases with the increase of branches, but the second-order molecular connectivity index (^2^ *χ*) increases with the increase of branches, whereas the valence molecular connectivity index of zero order (^0^ *χ* ^*ν*^) encodes the information about heteroatoms [10, 22]. Thus, we keep these indices in the next considerations. These indices define models 1–3 in the first step. To these indices, the remaining indices were added separately (step 2). The values of the correlation coefficients for this step (second step) are contained in Table 4.

**Table 4:** Values of correlation coefficients for models 1–3 in step 2

Indices	^0^ *χ*	^1^ *χ*	^2^ *χ*	^3^ *χ*_*c*_	^4^ *χ*_*pc*_	^0^ *χ* ^*ν*^	^1^ *χ* ^*ν*^	^2^ *χ* ^*ν*^	${}^3\chi_c^{\nu }$	${}^4\chi_{pc}^{\nu }$
Model 1	0.982	0.982	–	0.982	0.983	0.982	0.982	0.982	0.982	0.982
Model 2	0.978	–	0.982	0.983	0.976	0.977	0.976	0.977	0.983	0.978
Model 3	0.979	0.977	0.982	0.978	0.975	–	0.978	0.976	0.976	0.975

Because the ^2^ *χ* index alone gives *r* = 0.982, therefore the relationships with pair of indices ^1^ *χ* and^2^ *χ* (*r* = 0.982) and also with the pair ^0^ *χ* ^*ν*^ and^2^ *χ* (*r* = 0.982) indices can be ignored in the further investigations. Next, from Table 4, it follows that in the case of models 1 and 3, the values of the correlation coefficients did not change significantly, so for those models, the step by step process was ended. In the case of model 2, the values of the correlation coefficients are higher for the relationships which contain additionally ^3^ *χ*_*c*_ or ${}^3\chi_c^{\nu }$ indices. The ^3^ *χ*_*c*_ index encodes the information about the number of branches and their environment [10, 22]. The ${}^3\chi_c^{\nu }$ index adds information about heteroatoms. Thus, the relationship containing the ${}^3\chi_c^{\nu }$ index is richer in structural information than with the ^3^ *χ*_*c*_ index. Furthermore, the addition others indices (step 3) did not change significantly the values of correlation coefficients therefore model 2 is now defined by the pair of indices ^1^ *χ*and ${}^3\chi_c^{\nu }$.

The obtained formulas (models 1–3) are given below:

7 $$ \mathrm{Model}\ 1: \log cmc=-0.17261-0.184411\cdot {}^2\chi $$

8 $$ \mathrm{Model}\ 2: \log cmc=0.18447-0.14866\cdot {}^1\chi -0.19248\cdot {}^3\chi_c^{\nu } $$

9 $$ \mathrm{Model}\ 3: \log cmc=0.44266-0.12317\cdot {}^0\chi^{\nu } $$

The statistical characteristics of the descriptors included in models 1–3 are shown in Appendix 2.

**Fig. 2:**
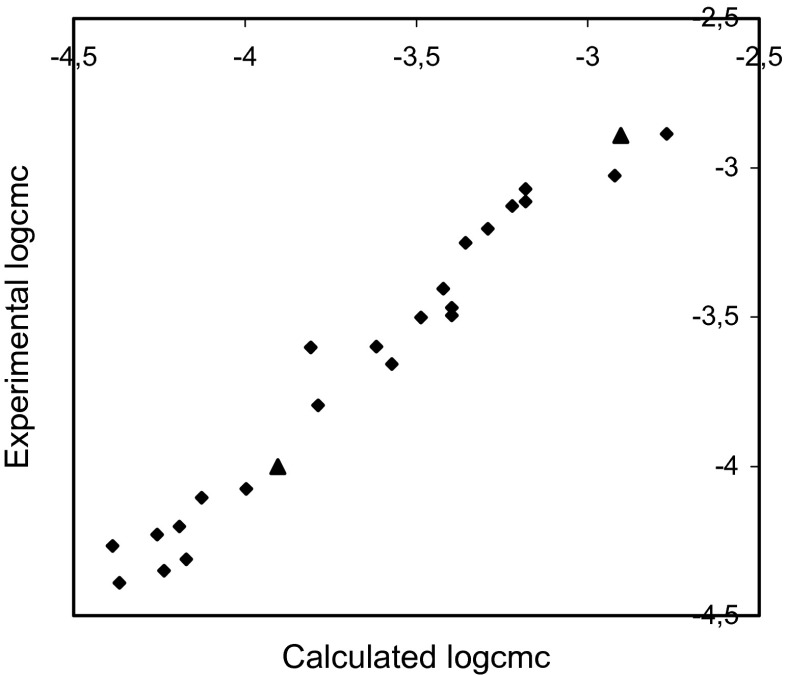
Plot of the experimental logcmc versus thatcalculated using Eq. 7 for training set (*rhomb*) (*r* = 0.982, *F* = 563.629, *s* = 0.095) and test compounds (*triangle*)

**Fig. 3:**
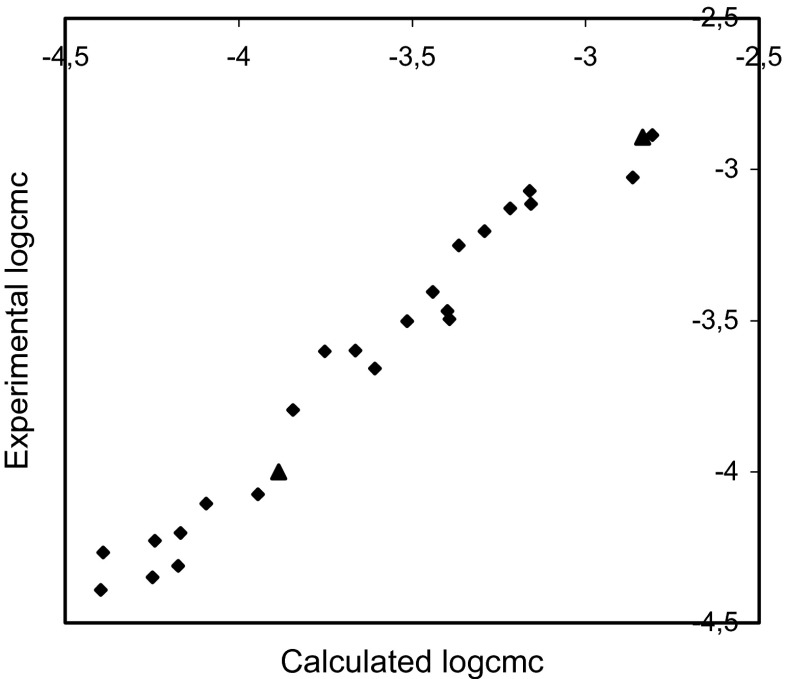
Plot of the experimental logcmc versus thatcalculated using Eq. 8 for training set (*rhomb*) (*r* = 0.983, *F* = 585.435, *s* = 0.093) and test compounds (*triangle*)

**Fig. 4:**
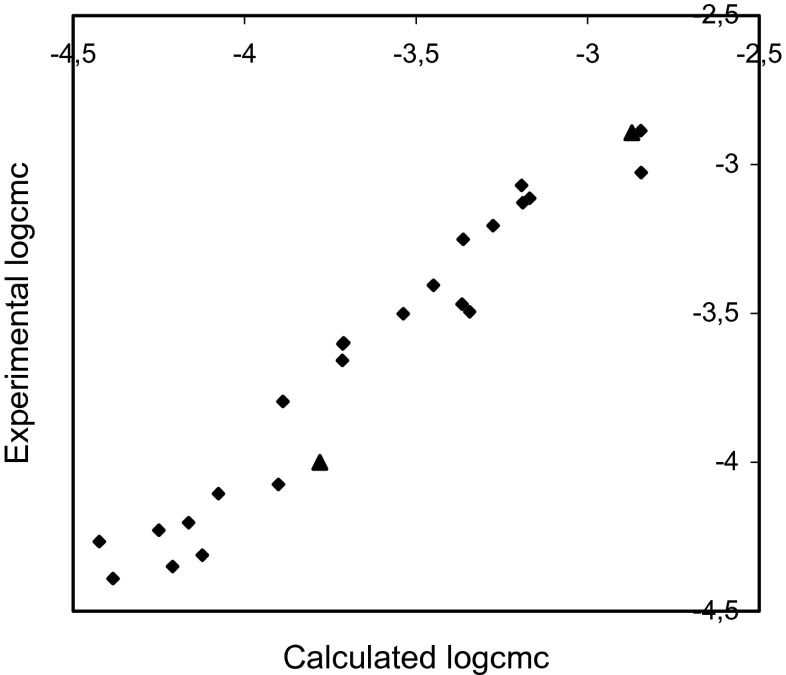
Plot of the experimental logcmc versus thatcalculated using Eq. 9 for training set (*rhomb*) (*r* = 0.975, *F* = 403.639, *s* = 0.111) and test compounds (*triangle*)

The plots of the experimental logcmc versus the logcmc calculated using Eqs. 7–9 are presented in Figs. 2, 3, and 4.

The comparisons of the experimental logcmc with the values calculated using Eqs. 7–9 presented in Figs. 2, 3, and 4 show that models 1–3 estimate the logcmc of compounds from the training set very well, and model 2 is slightly better than model 1 and better than Model 3. The values of coefficients of determination are equal to 0.964, 0.966, and 0.951 for models 1, 2, and 3, respectively.

**Fig. 5:**
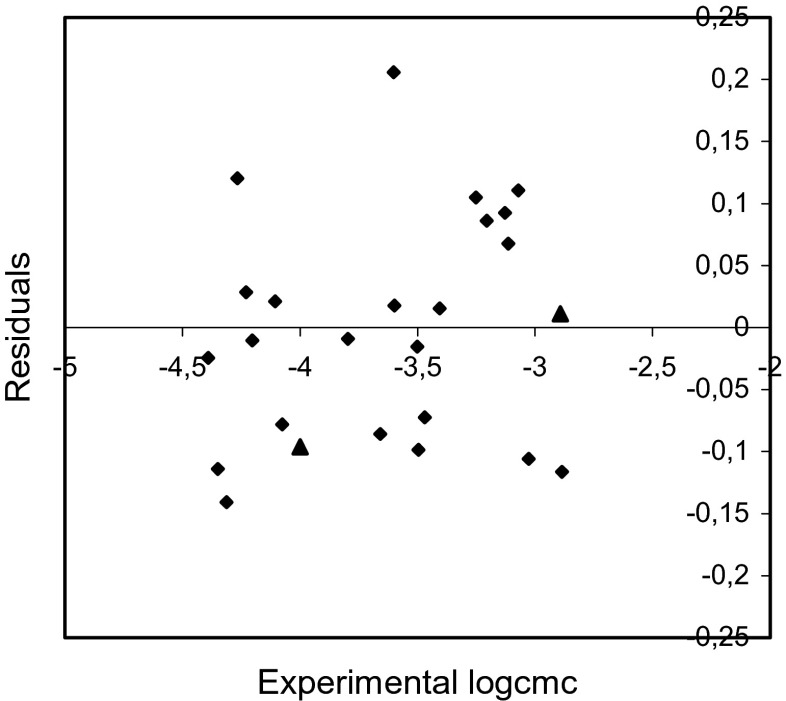
Plot of residuals versus the experimental logcmc values for training set (*rhomb*) and test compounds (*triangle*) (model 1)

**Fig. 6:**
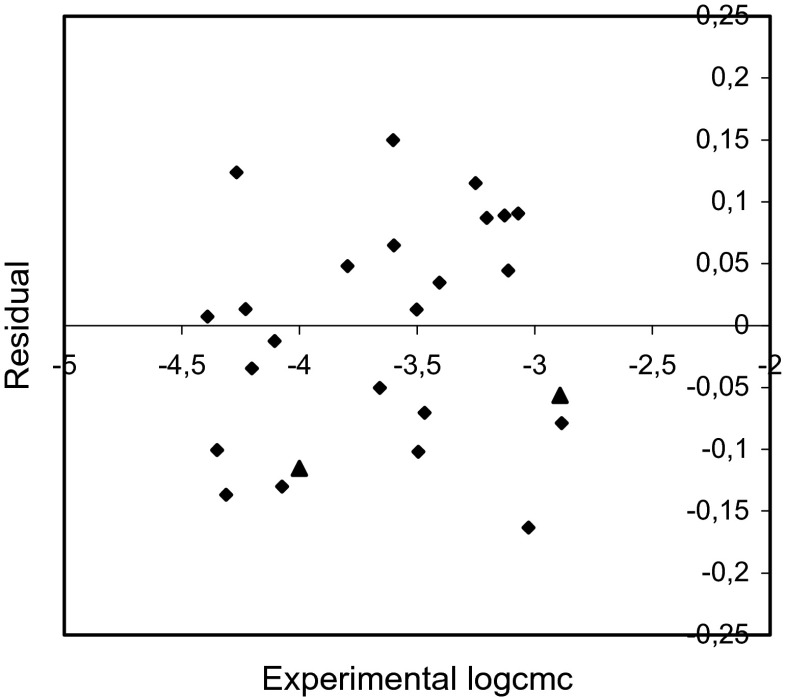
Plot of residuals versus the experimental logcmc values for training set (*rhomb*) and test compounds (*triangle*) (model 2)

**Fig. 7:**
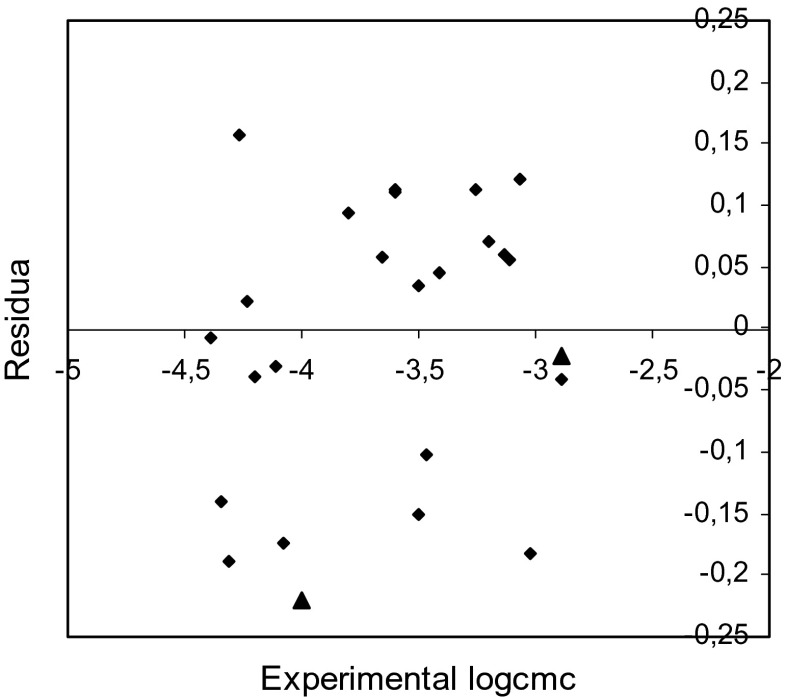
Plot of residuals versus the experimental logcmc values for training set (*rhomb*) and test compounds (*triangle*) (model 3)

The plots of residuals versus the experimental values of logcmc are shown in Figs. 5, 6, and 7.

The examination of the residuals (Figs. 5, 6, and 7) shows generally good agreement between the experimental and calculated values of logcmc. Most of the residuals are close to zero and only one residual for model 1 is slightly larger than 2*s*.

The obtained models were used to estimate the logcmc values of other compounds, different from gemini surfactants from the training set. The values of the literature logcmc for test compounds are listed in Table 5.

**Table 5:** Experimental logcmc values [20, 21] of test compounds

Compound^a^	Experimentallogcmc
12-Py(2)-4-(2)Py-12⋅2Cl^−^	−2.89279
p-[C_14_H_29_N^+^(CH_3_)_2_CH_2_CH(OH)CH_2_O]_2_C_6_H_4_⋅2Cl^−^	−4.0

^a^The structures of the compounds are presented in Fig. 1

The comparison of the experimental values of logcmcof the compounds used in the test with the values estimated using Eqs. 7–9 is shown in Figs. 2, 3, and 4. The agreement between predicted and experimental logcmc values of the test compounds is very good. The plots of residuals (Figs. 5, 6, and 7) confirm this agreement.

In brief, the best model in the first step is that which contains the second-order molecular connectivity index (^2^ *χ*) (model 1). The second step shows that the relationship containing the first-order molecular connectivity index (^1^ *χ*) and the third-order cluster valence molecular connectivity index (${}^3\chi_c^{\nu }$) (model 2) estimates slightly better the values of the critical micelle concentration of cationic (chloride) gemini surfactants.

The second-order molecular connectivity index (^2^ *χ*) appearing in model 1 does not differentiate heteroatoms;it represents two-bond terms within the molecule and its values depend on the isomers of the compound [10]. The values of ^2^ *χ* index increase with the increase in length and branches of hydrocarbon chains. The zeroth-order valence molecular connectivity index (^0^ *χ* ^*ν*^) appearing in model 3 relates to the atoms of the molecule, and it differentiates heteroatoms. The values of ^0^ *χ* ^*ν*^ index increase with the increase in length and branches of hydrocarbon chains, and its values are smaller for the compounds containing in their structure heteroatoms in comparison with those of their hydrocarbon analogous compounds. The first-order molecular connectivity index (^1^ *χ*) appearing in model 2 does not differentiate heteroatoms;it represents the one-bond terms within the molecule. The values of ^1^ *χ* index depend on the isomers of the compound and, in this case, decrease with the increase in branches, but its values increase with the increase in length of hydrocarbon chains. The third-order cluster valence molecular connectivity index (${}^3\chi_c^{\nu }$) appearing in model 2 represents three-bond cluster terms within the molecule, and it differentiates heteroatoms. The values of ${}^3\chi_c^{\nu }$ index increase with the increase in branches of hydrocarbon chains, and its values are smaller for the compounds containing in their structure heteroatoms in comparison with those of their hydrocarbon analogous compounds. All models contain the molecular connectivity indices with negative coefficients, thus as their values increase, the cmc decreases.

So, from Eqs. (7–9) and also from Table 1, it follows that as the number of methylene groups increases in the hydrocarbon chains,the cmc decreases. For example, for compound bis(EA-m-3iso)C6 (*m* = 9, 11), the experimental values of cmc are the following: 0.084 and 0.059 mM[], and the calculated values of cmc are the following: 0.101and 0.055 mM (model 1), 0.113and 0.057 mM (model 2), and 0.125and 0.056 mM (model 3). Also, as the number of methylene groups increases in the spacer group then the experimental and also the calculated values of the cmc decrease. For example, for compound AC_12_C_n_C_12_A (*n* = 6, 12), the experimental values of cmc are the following: 0.340and 0.160 mM[18], and the calculated values of cmc are the following: 0.401and 0.163 mM (model 1), 0.399and 0.143 mM (model 2), and 0.430and 0.129 mM (model 3). In the case of compounds bis(EA-m-3)R and bis(EA-m-3iso)R for *R* = 5, 6, 8, the experimental and also the calculated values of cmc decrease too with the increase in the alkyl chain length at the central nitrogen atom in the molecule. Thus, the increase in length of hydrocarbon chain and simultaneously in flexibility of this chain results in the decrease of cmc values.

The comparison of the compounds with straight and branched chains shows that the branches differently influence the calculated cmc values. For example, for compounds bis(EA-11-3)C8 and bis(EA-11-3iso)C8 using model 1, we obtain the following values of cmc: 0.043and 0.041 and the following using model 3: 0.041and 0.038 mM, whereas using model 2, we obtain 0.040and 0.041 mM, respectively. The experimental cmc values are 0.041 mM for compound bis(EA-11-3)C8 and 0.054 mM for compound bis(EA-11-3iso)C8 [17]. It means that the experimental value of cmc is higher for the compound bis(EA-11-3iso)C8; therefore, the cmc values calculated using model 2 are in good agreement with the experimental results. Some othergemini surfactants and the corresponding calculated values of logcmcare presented in Appendix 3. For the compounds presented in in Appendix 3, the cmc values which are calculated using models1 and 2 are smaller for the compounds with branched chains than for those with straight chains and the same number of atoms. Using model 3, the cmc values are smaller only for compounds with branched carbon chains but for compounds containing heteroatoms, the branches cause the higher cmc values. The result obtained for the compounds containing heteroatoms is in agreement with the experimental one [23]. That is, for chloride compounds C_12_EO_1_C_12_ (0.5 mM at 20 °C) and C_12_C_4_(OH)C_12_ (0.65 mM at 20 °C) [23].

The comparison of the heteroatom compounds with their hydrocarbon analogous compounds (Appendix 3) shows that the presence of heteroatoms in the molecules results in higher calculated, using Model 3, values of critical micelle concentration in comparison with its carbon analogous compounds. Model 2 differentiates heteroatoms only on branches but Model 1 does not differentiate heteroatoms. Some experimental results show higher values of critical micelle concentration of gemini surfactants containing in their structure heteroatoms in comparison with those of their hydrocarbon analogous compounds [18, 24–26]. That is, for example, for bromide compounds C_12_EO_2_C_12_ (1.09 mM[24]) and C_12_C_8_C_12_ (0.84 mM[25]) and also for C_12_7NHC_12_ (1.17 mM[26] and 1.21 mM[18]) and C_12_C_7_C_12_ (0.9 mM[26]). Also, the theoretical results obtained for cationic (bromide) gemini surfactants with various spacer group only [13] show that the presence of heteroatoms in the spacer group results in higher value of cmc. Thus,model 3 better describes the effect of heteroatoms on cmc values.

In brief, the investigated models (models 1–3 ) show high correlations between logcmc and the molecular connectivity indices and statistically, the best models (models1–2) can be used to estimate the values of critical micelle concentration, but the description of the effect of the structure of investigated compounds on cmc values by those models is different. All models describe the cmc values very well if we take into account only the elongation of alkyl chains. In the case of branches and heteroatoms, these models differently describe cmc values and some results differ from the experimental ones. It suggests that another index will be better to describe the effect of the structure on critical micelle concentration of cationic (chloride) gemini surfactants. Because some experimental data show that the branched chains especially branched hydrocarbon chains [17, 23, 27], and also heteroatoms [24–26], cause the higher cmc values therefore the best index which will satisfactorilydescribe the effect of the chemical structure on cmc value is the first-order valence molecular connectivity index (^1^ *χ* ^*ν*^). The first-order valence molecular connectivity index (^1^ *χ* ^*ν*^) is similar to the first-order molecular connectivity index (^1^ *χ*), but it includes heteroatom information.The values of ^1^ *χ* ^*ν*^ index increase with the increase in length of hydrocarbon chains, and its values decrease with the increase in branches. This index differentiates heteroatoms and its values are smaller than the values of the ^1^ *χ* index.

The formula containing the ^1^ *χ* ^*ν*^ index is the following:

10 $$ \mathrm{Model}\ 4: \log cmc=0.56045-0.20443\cdot {}^1\chi^{\nu } $$

The statistical characteristic of the selected descriptor is given in Appendix 2.

From Eq. 10, it follows that as the number of methylene groups increases in the hydrocarbon chains and also in spacer chain,the cmc decreases. For example, for compound bis(EA-m-3iso)C8 (*m* = 9, 11), the experimental values of cmc are the following: 0.078and 0.054 mM[17], and the calculated values of cmc are the following: 0.104and 0.040 mM. For compounds with different spacer lengths AC_12_C_n_C_12_A (*n* = 6, 12), the experimental values of cmc are the following: 0.340and 0.160 mM[18], and the calculated values of cmc are the following: 0.414and 0.101 mM, respectively. The comparison of the compounds with straight and branched chains shows that the calculated values are also in good agreement with the experimental results. The example arethe compoundsbis(EA-11-3)C8 and bis(EA-11-3iso)C8, for which the experimental cmc values are the following: 0.041and 0.054 mM, and the calculated using model 4 cmc values are the following: 0.038and 0.040 mM, respectively.

**Fig. 8:**
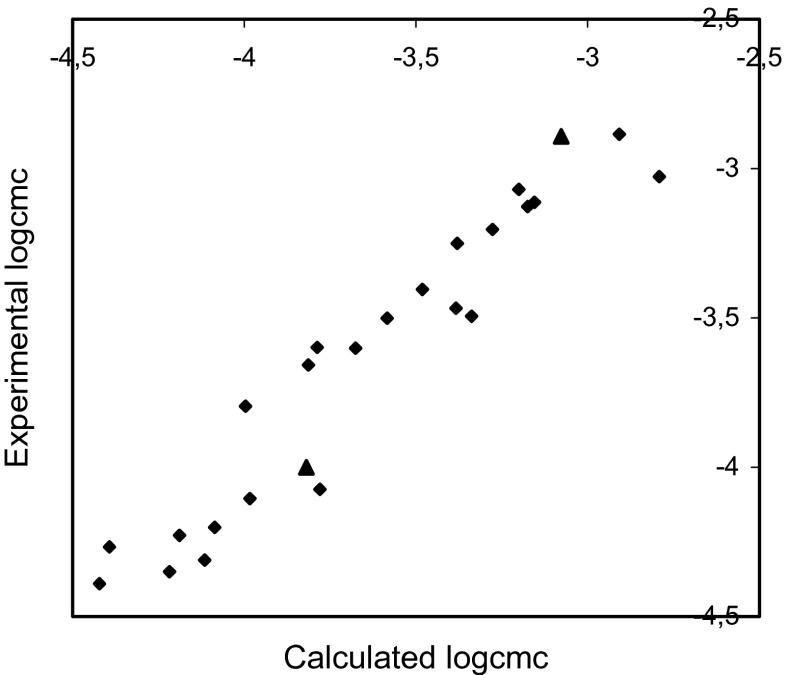
Plot of the experimental logcmc versus thatcalculated using Eq. 10 for training set (*rhomb*) (*r* = 0.957, *F* = 231.36, *s* = 0.144) and test compounds (*triangle*)

**Fig. 9:**
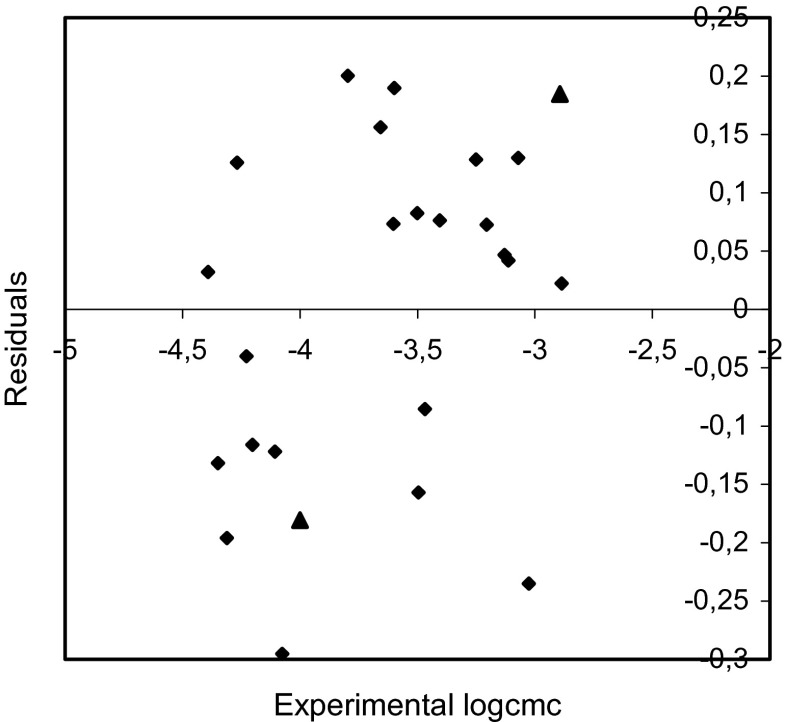
Plot of residuals versus the experimental logcmc values for training set (*rhomb*) and test compounds (*triangle*) (model 4)

The plot of the experimental logcmc versus the logcmc calculated using Eq. 10 and the plot of residuals versus the experimental values of logcmc for training set and test compounds are shown in Figs. 8 and 9

The statistical parameters show that model 4 estimates logcmc values of investigated compounds lower than models 1–3, but comparison of the experimental and calculated values of cmc by means of the effect of the structural elements on cmc values shows that the values of critical micelle concentration calculated using model 4 are in good agreement with the experimental results. Some additional comparisons are presented in Appendix 3.

The data contained in Appendix 3 show that the increase in the number of atoms by lengthening or by the increase of branches causes the decrease of the cmc value calculated using models1–4. If we take in to account the heteroatom compounds, the effect of branches is in good agreement with experimental results obtained for bromide compounds [28]. But in the case of the elongation of hydrophilic spacer, as is for compounds C_12_EO_n_C_12_, the experimental results [24] show the opposite behavior. Maybe it is due to the fact that the length of hydrocarbon chains has the dominant effect on cmc values and, in consequence, on obtained models.

The experimental studies [18] show also that the cmc values of chloride gemini surfactants are higher than of bromides ones. Indeed, the experimental cmc values of C_12_C_6_C_12_gemini surfactant with bromides and chlorides as counterions are the following: 0.89and 1.30 mM[18], respectively. But, using previous model [12] for bromide geminis and present (Eqs. 7–10) for chlorides ones, we obtain the following calculated values of cmc: 1.11 mM[12] and 1.70 mM (model 1), 1.56 mM (model 2), 1.43 mM (model 3), and 1.24 mM (model 4), respectively. So, both the experimental and the calculated values of cmc of cationic (chloride) gemini surfactants are higher than for the bromide ones and in the case of chloride surfactants, the best estimated value is for model 4.

It is worth to add that the test compounds (Table 5) differ in structure of spacer and head groups from the training set compounds, but also for those molecules, the agreement between predicted and experimental logcmc values is very good.

## Conclusion

In the present work, the cationic (chloride) gemini surfactants with various structures were taken into account. All the models obtained confirm the experimental results that the length of alkyl chains plays the major role in micelle formation. The present study shows that although the second-order molecular connectivity index correlates high with logcmc values of cationic gemini surfactants, the statistically lower correlation logcmc with the first order valence molecular connectivity index better describes the effect of the branches and heteroatoms on the critical micelle concentration of cationic (chloride) gemini surfactants. Becausemodel 4 (Eq. (10)) has good prediction ability of investigated compounds,it can be used to predict the critical micelle concentration and in particular to design new cationic (chloride) gemini surfactants more active in micelle formation.

## Figures and Tables

**Fig. 10:**
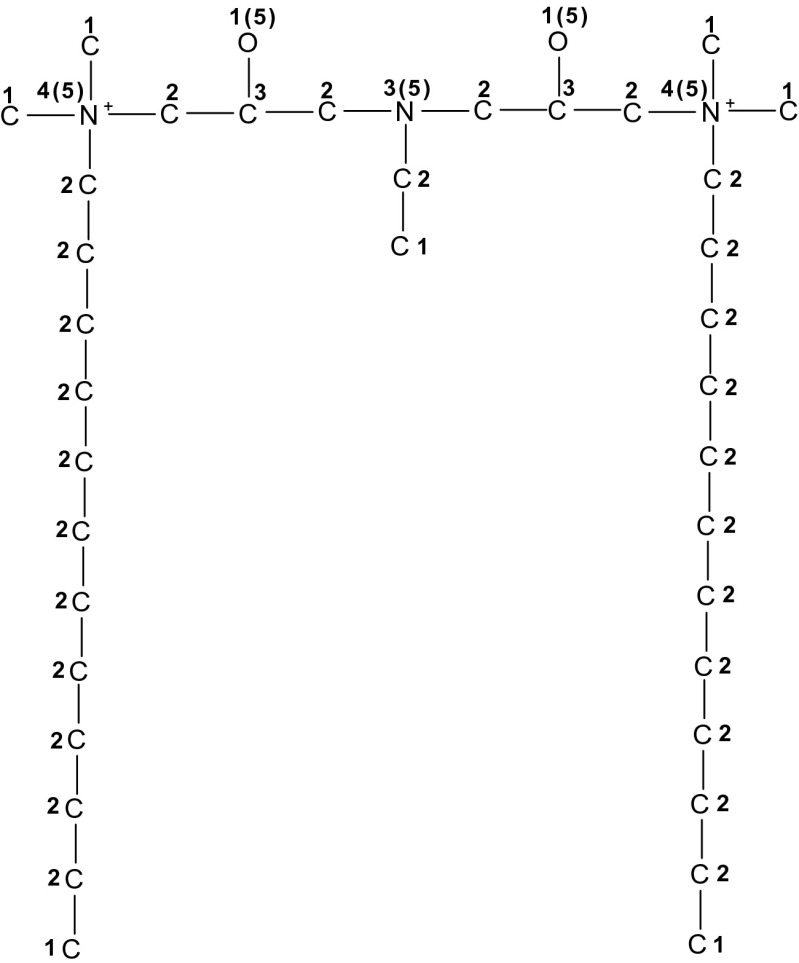
Hydrogen-suppressed graphic structural formula of exemplary gemini surfactant anddelta values

**Table 7:**
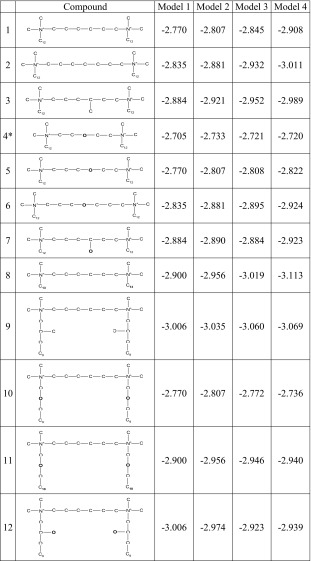
Hydrogen-suppressed structural formulas of some gemini surfactants and calculated logcmc values

^a^For this compound, the experimental values of cmc are 0.5 (mM) at 20 °C [23] and 2.2 (mM) at about 23 °C [29] and the calculated values of cmc using models1–4 are about 1.9 (mM) for 25 °C

## Appendix 1

To illustrate the calculation of the molecular connectivity indices, the 2agemini surfactant (Table 1) was taken into account. The first step of calculations is to draw the structural formula of the molecule and to count the values of connectivity degrees [10]. The hydrogen atoms are suppressed in graphic structural formula. The structure along with the values of connectivity and valence connectivity degrees are shown in Fig. 10.

Next, the molecule is dissected into the appropriate fragments, for example: path, cluster, or path-cluster. The values of connectivity indices can be easily calculate using Eq. 1.

The calculations of molecular connectivity indices for exemplary gemini surfactant read:

$$ \begin{array}{l}{}^0\chi ={\displaystyle \sum {\left({\delta}_i\right)}^{-0.5}=}\\ {}=9\cdot {(1)}^{-0.5}+27\cdot {(2)}^{-0.5}+2\cdot {(4)}^{-0.5}+3\cdot {(3)}^{-0.5}=\\ {}=30.82393\\ {}{}^1\chi ={\displaystyle \sum {\left({\delta}_i\times {\delta}_j\right)}^{-0.5}}=\\ {}=3\cdot {\left(2\times 1\right)}^{-0.5}+20\cdot {\left(2\times 2\right)}^{-0.5}+4\cdot {\left(4\times 1\right)}^{-0.5}+4\cdot {\left(2\times 4\right)}^{-0.5}+7\cdot {\left(2\times 3\right)}^{-0.5}+2\cdot {\left(3\times 1\right)}^{-0.5}=\\ {}=19.54797\\ {}{}^2\chi ={\displaystyle \sum {\left({\delta}_i\times {\delta}_j\times {\delta}_k\right)}^{-0.5}=}\\ {}=2\cdot {\left(2\times 2\times 1\right)}^{-0.5}+18\cdot {\left(2\times 2\times 2\right)}^{-0.5}+4\cdot {\left(2\times 2\times 4\right)}^{-0.5}+8\cdot {\left(4\times 2\times 1\right)}^{-0.5}+2\cdot {\left(1\times 4\times 1\right)}^{-0.5}+\\ {}+2\cdot {\left(4\times 2\times 3\right)}^{-0.5}+5\cdot {\left(2\times 3\times 1\right)}^{-0.5}+5\cdot {\left(2\times 3\times 2\right)}^{-0.5}+2\cdot {\left(3\times 2\times 3\right)}^{-0.5}=\\ {}=16.55666\\ {}{}^3\chi_c={\displaystyle \sum {\left({\delta}_i\times {\delta}_j\times {\delta}_k\times {\delta}_l\right)}^{-0.5}=}\\ {}=4\cdot {\left(4\times 2\times 2\times 1\right)}^{-0.5}+4\cdot {\left(4\times 2\times 1\times 1\right)}^{-0.5}+2\cdot {\left(2\times 3\times 2\times 1\right)}^{-0.5}+{\left(2\times 3\times 2\times 2\right)}^{-0.5}=\\ {}=3.19569\\ {}{}^4\chi_{pc}={\displaystyle \sum {\left({\delta}_i\times {\delta}_j\times {\delta}_k\times {\delta}_l\times {\delta}_m\right)}^{-0.5}=}\\ {}=2\cdot {\left(1\times 4\times 2\times 2\times 1\right)}^{-0.5}+4\cdot {\left(1\times 4\times 2\times 2\times 2\right)}^{-0.5}+2\cdot {\left(1\times 4\times 1\times 2\times 3\right)}^{-0.5}+6\cdot {\left(1\times 4\times 2\times 2\times 3\right)}^{-0.5}+\\ {}+2\cdot {\left(1\times 3\times 2\times 2\times 3\right)}^{-0.5}+2\cdot {\left(2\times 3\times 2\times 2\times 3\right)}^{-0.5}+{\left(2\times 3\times 2\times 2\times 1\right)}^{-0.5}=\\ {}=3.25454\end{array} $$

and

$$ \begin{array}{l}{}^0\chi^{\nu }={\displaystyle \sum {\left({\delta}_i^{\nu}\right)}^{-0.5}=}\\ {}=7\cdot {(1)}^{-0.5}+27\cdot {(2)}^{-0.5}+2\cdot {(3)}^{-0.5}+5\cdot {(5)}^{-0.5}=\\ {}=29.48265\\ {}{}^1\chi^{\nu }={\displaystyle \sum {\left({\delta}_i^{\nu}\times {\delta}_j^{\nu}\right)}^{-0.5}}=\\ {}=3\cdot {\left(2\times 1\right)}^{-0.5}+20\cdot {\left(2\times 2\right)}^{-0.5}+7\cdot {\left(2\times 5\right)}^{-0.5}+4\cdot {\left(5\times 1\right)}^{-0.5}+4\cdot {\left(2\times 3\right)}^{-0.5}+2\cdot {\left(5\times 3\right)}^{-0.5}=\\ {}=18.27316\\ {}{}^2\chi^{\nu }={\displaystyle \sum {\left({\delta}_i^{\nu}\times {\delta}_j^{\nu}\times {\delta}_k^{\nu}\right)}^{-0.5}=}2\cdot {\left(2\times 2\times 1\right)}^{-0.5}+18\cdot {\left(2\times 2\times 2\right)}^{-0.5}+7\cdot {\left(2\times 2\times 5\right)}^{-0.5}+2\cdot {\left(1\times 5\times 1\right)}^{-0.5}+\\ {}+9\cdot {\left(1\times 5\times 2\right)}^{-0.5}+8\cdot {\left(5\times 2\times 3\right)}^{-0.5}+2\cdot {\left(2\times 2\times 3\right)}^{-0.5}=\\ {}=14.70763\\ {}{}^3\chi_c^{\nu }={\displaystyle \sum {\left({\delta}_i^{\nu}\times {\delta}_j^{\nu}\times {\delta}_k^{\nu}\times {\delta}_l^{\nu}\right)}^{-0.5}=}\\ {}=4\cdot {\left(5\times 2\times 2\times 1\right)}^{-0.5}+4\cdot {\left(5\times 2\times 1\times 1\right)}^{-0.5}+2\cdot {\left(5\times 2\times 2\times 3\right)}^{-0.5}+{\left(5\times 2\times 2\times 2\right)}^{-0.5}=\\ {}=2.57565\\ {}{}^4\chi_{pc}^{\nu }={\displaystyle \sum {\left({\delta}_i^{\nu}\times {\delta}_j^{\nu}\times {\delta}_k^{\nu}\times {\delta}_l^{\nu}\times {\delta}_k^{\nu}\right)}^{-0.5}=}\\ {}=2\cdot {\left(1\times 5\times 2\times 2\times 1\right)}^{-0.5}+5\cdot {\left(1\times 5\times 2\times 2\times 2\right)}^{-0.5}+2\cdot {\left(1\times 5\times 2\times 3\times 1\right)}^{-0.5}+4\cdot {\left(1\times 5\times 2\times 2\times 3\right)}^{-0.5}+\\ {}+4\cdot {\left(3\times 5\times 2\times 2\times 5\right)}^{-0.5}+2\cdot {\left(2\times 5\times 2\times 2\times 3\right)}^{-0.5}=\\ {}=2.53284\end{array} $$

## Appendix 2

The statistical characteristics of the descriptors contained in models1–4 are given in Table 6.

**Table 6:** Characteristics of descriptors

Model	Constant/descriptor	Coefficient	Standard error	*t* value	*p* value
1	Constant ^2^ *χ*	−0.17261 −0.18411	0.14814 0.00775	−1.165 −23.741	0.257006 0.000000
2	Constant ^1^ *χ* ${}^3\chi_c^{\nu }$	0.18447 −0.14866 −0.19248	0.16798 0.00845 0.06861	1.0982 −17.587 −2.806	0.285164 0.000000 0.010922
3	Constant ^0^ *χ* ^*ν*^	0.44266 −0.12317	0.20543 0.00613	2.155 −20.091	0.042935 0.000000
4	Constant ^1^ *χ* ^*ν*^	0.56045 −0.20443	0.27897 0.01344	2.009 −15.211	0.057569 0.000000

High absolute Student *t* values of the descriptors express that the regression coefficients of the descriptors are significantly larger than the standard error. Descriptors with *p* values below 0.05 (95 % confidence) are considered statistically significant [4].

As follows from Table 6, all the descriptors are statistically significant.

## Appendix 3

The hydrogen-suppressed graphic structural formulas of some gemini surfactants, and the corresponding calculated, using Eqs. 7–10, logcmc values are contained in Table 7.

.
